# Needs and preferences of patients regarding atopic dermatitis care in the era of new therapeutic options: a qualitative study

**DOI:** 10.1007/s00403-021-02321-z

**Published:** 2022-02-02

**Authors:** Linde E. M. de Wijs, Sven van Egmond, Arjan C. A. Devillers, Tamar Nijsten, DirkJan Hijnen, Marjolein Lugtenberg

**Affiliations:** 1 Department of Dermatology, Erasmus MC University Medical Center, Dr. Molewaterplein 40, 3015 GD Rotterdam, The Netherlands; 2 Department of Dermatology, Maasstad Hospital, Rotterdam, The Netherlands

**Keywords:** Atopic dermatitis, Atopic eczema, Therapy, Qualitative research, Patient-centered care

## Abstract

To optimally tailor atopic dermatitis (AD) care to patients’ needs, especially considering the many emerging therapeutic options, insight into patients’ needs and preferences regarding AD care is needed. To explore patients’ needs and preferences regarding AD care, a qualitative study consisting of three focus groups with a total of 20 adult AD patients was conducted. All sessions were transcribed verbatim and inductively analyzed using several phases of coding to create an overview of patients’ needs and preferences. AD patients emphasized the need for a patient-tailored approach in all identified aspects of AD care. With regard to consultations, patients stressed the need for a personal approach and increased recognition of the disease impact, which should mainly be determined by patients. With regard to the organization of AD care, the need for psychosocial and medical supportive care as well as quick access to health-care providers during disease flares was emphasized. Within the decision-making process, patients indicated that the provided information, the role of the patient and physician, whether or not treatment goals should be set, and decisive factors for indication and feasibility of novel therapies should be patient dependent. AD care should be patient tailored with increased attention for the psychosocial burden, as well as better access to health care during disease flares. To provide patient-tailored care, the personal situation, needs, and preferences of the patient should be taken into account in the therapeutic decision-making process, with respect for the autonomy of the patient.

**Supplementary Information:**

The online version contains supplementary material available at 10.1007/s00403-021-02321-z.

## Introduction

Atopic dermatitis (AD) is a chronic inflammatory skin disease, characterized by a relapsing and remitting disease course. AD-related symptoms including itch, pain and insomnia are known to have a large impact on quality of life [1–4]. Better acceptance and recognition of the emotional and psychological burden associated with AD are among the most important wishes of AD patients [3].

AD treatment is mainly focused on medicinal therapy, usually starting with topical treatment including emollients, topical corticosteroids, and calcineurin inhibitors [5]. Until recently, treatment of patients with moderate-to-severe AD was limited to phototherapy or conventional systemic immunosuppressants. However, the number of therapeutic options available to physicians has increased rapidly in the last few years [6]. At the same time, patients’ preferences, standards, and expectations are changing and play an increasing role in the therapeutic decision-making process [7, 8].

Experiences, needs, and preferences regarding specific topics (e.g., autonomy, influence of (social) media, influence on family life or financial impact) have been assessed, mainly among adolescents or parents of pediatric AD patients [1–3, 9–11]. However, a complete overview concerning all aspects of today’s AD care is needed to adequately tailor AD care to patients’ needs, especially in times of many emerging therapies. Therefore, the aim of this study was to explore patients’ needs and preferences regarding AD care.

## Methods

A qualitative study consisting of three focus groups was conducted to obtain an in-depth understanding of patients’ views on AD care. Qualitative research is particularly suitable as it primarily focuses on interpretations, rather than quantification of patients’ beliefs, emotions, behaviors, and interactions in daily life [12]. Additionally, the interactive and dynamic process of focus groups attributes to identification and clarification of patients’ views, leading to richer and more diverse information in comparison to individual interviews [13, 14]. This focus group study was designed and is reported in accordance with the SRQR recommendations. [15].

### Study setting and participants

This study was conducted at the Erasmus MC University Medical Center (academic hospital). Purposive sampling was used to obtain a variable sample of AD patients in terms of age, sex, and therapeutic experiences [14, 16, 17]. Eligible patients were recruited at the outpatient clinic of the Erasmus MC and Maasstad Hospital (general hospital, Rotterdam) and received a study information leaflet. Patients could sign up by contacting the researchers. They were offered a voucher of €40 for participating. After including three focus groups with a total of 20 participants (6–8 participants per focus group), we concluded that data saturation was reached and recruitment of participants ended.

### Data collection

Prior to the start of the focus groups, patients provided written informed consent and completed a patient characteristics questionnaire. After we made sure that all participants felt able to share their views and experiences, the focus groups were started, structured by a topic guide (see Online Resource 1), which was based on researchers’ experiences, literature, and therapy guidelines [18, 19]. Discussions were moderated by an experienced moderator of focus groups (ML or SE) and a physician with extensive experience in treating AD patients, but not involved in direct patient care of the participants (LW). All focus groups were audiotaped and transcribed verbatim.

### Data analysis

**Fig. 1:**
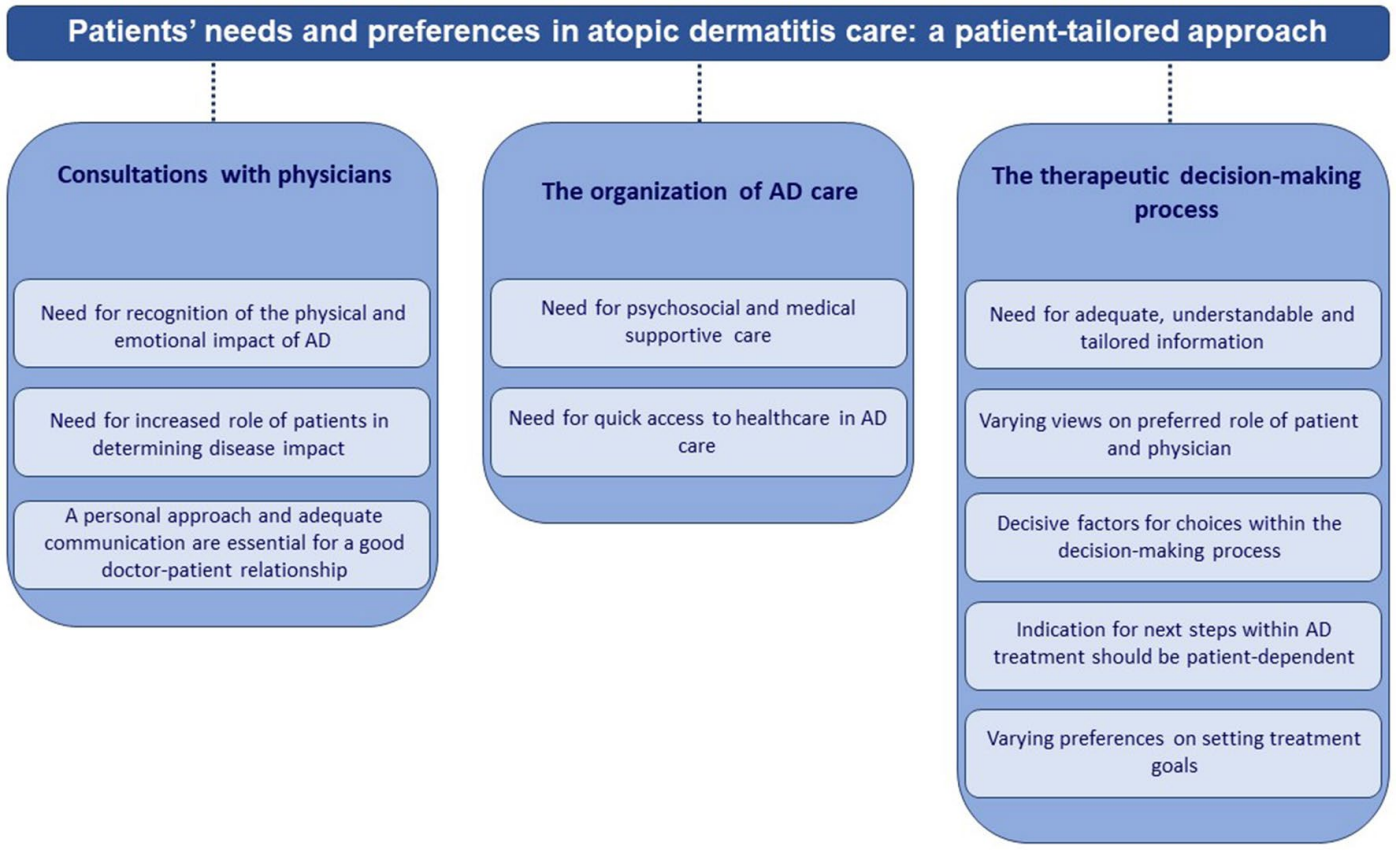
Overview of needs and preferences of patients in AD care. Needs and preferences regarding several topics withing three main aspects of AD care were identified

The transcripts were analyzed by using Nvivo version 12 plus. An inductive approach to data analysis was applied using several phases of coding (guided by a codebook) combined with the constant comparison technique (comparing emerged concepts with new data and across groups) [20]. First, the transcripts were thoroughly read and roughly summarized by one researcher (LW) to get a holistic understanding of the focus groups. Subsequently, the first two transcripts were openly coded (i.e., inductively and line by line) [20] by one researcher (LW) and independently checked by another (ML). This resulted in a list of unstructured codes. More abstract and structured concepts of relevance were obtained through axial coding [20]. Using this structured coding scheme, the transcript of the third focus group was coded (LW) and independently checked (SE). No new concepts emerged during coding of the third focus group and data saturation was reached. The identified concepts and their subcategories were discussed during several multidisciplinary (psychologist, dermatologist and PhD student) research team meetings. This resulted in an overview of core needs and preferences within three central aspects of AD care (see Fig. 1).

## Results

### Patient characteristics

Table 1 and Online Resource 2 provide sample characteristics and demographics.

**Table 1:** Participants’ characteristics

Setting	Focus group 1	Focus group 2	Focus group 3	Total
	Erasmus MC	Maasstad Hospital	Erasmus MC	
Participants—*n*	8	6	6	20
Age (years)				
Median (IQR)	34 (20–48)	28 (27–57)	33 (31–40)	31 (24–48)
Min, max	19, 63	21, 62	20, 63	19, 63
Female—*n* (%)	5 (64)^a^	4 (67)	2 (33)	11 (55)
Current treatment—*n* (%)				
Topical	1 (12.5)	2 (33)	1 (17)	4 (20)
Systemic immunosuppressant	5 (63)	4 (67)	2 (33)	11 (55)
Biologic	2 (25)	0 (0)	3 (50)	5 (25)
Self-reported impact of disease—*n* (%)				
Very low	1 (12.5)	0 (0)	2 (33)	3 (15)
Low	2 (25)	0 (0)	0 (0)	2 (10)
Neutral	1 (12.5)	2 (33)	0 (0)	3 (15)
High	2 (25)	1 (17)	1 (17)	4 (20)
Very high	2 (25)	3 (50)	3 (50)	8 (40)

*IQR* interquartile range

^a^*n* = 1 transgender man

### Patients’ needs and experiences in AD care: a patient-tailored approach

Several needs and preferences were identified within three main aspects of AD care (Fig. 1): consultations with physicians, organization of AD care, and the therapeutic decision-making process. These needs and preferences are described in detail below. Additional illustrative quotes can be found in Online Resource 3. The need for a patient-tailored approach emerged as an overarching theme in all aspects of AD care. Patients stressed the need to better take patients’ characteristics and personal needs and preferences into account throughout AD care.

### The consultations with physicians

#### Need for recognition of the physical and emotional impact of AD

Patients reported that the major impact on both physical and emotional well-being of AD is sometimes underestimated by physicians. Patients emphasized the need for an increased recognition of the total burden and chronic pattern of their disease. Receiving empathy and being taken seriously were reported to be crucial in AD care.

*‘The moment you tell your physician that this is a real issue in your life, that it really is all-consuming, and your physician responds with “oh well, it’s not that bad”. It feels like a slap in the face.’*- focus group (FG) 1

Patients identified a need for physicians to pay particular attention to certain feelings (e.g., shame, loneliness, stress, and fear) and behaviors (e.g., social avoidance and poor sleeping behavior) associated with AD. Patients prefer their physician to take the initiative in talking about the impact of the disease, as they often feel uncomfortable to do so themselves (e.g., because of the busy schedules of physicians).

#### Need for increased role of patients in determining disease impact

Patients mentioned that physicians should take into account that the consultation is only a snapshot, which complicates the assessment of a relapsing disease. Patients highlighted the need to be able to show the eczema at its worst, for which patients use tools including writing down symptoms or photographing lesions during exacerbations. However, when discussing the severity of their disease in times of remission, they often feel unheard.

*‘AD does not only feel like a struggle against your own body, but it is also a struggle to be taken seriously and to prove that you are eligible for certain therapies instead of being sent home with another ointment.’* – FG 3

Patients expressed the need for an increased role of patients in determining disease impact. Most patients agreed that the treating physician is responsible for the evaluation of the medical situation (e.g., giving instructions, discussing treatment options, and laboratory tests). However, atopic dermatitis does not stop when the patients leave the clinic and patients indicated that they themselves are responsible for evaluating the disease impact in daily life. As such, patients highlighted that a physician’s respect for their autonomy in assessing the impact on their daily life would be appreciated.

#### A personal approach and adequate communication are essential for a good doctor–patient relationship

Patients indicated that the relationship between patient and physician plays an important role in their perceived quality of AD care. Patients prefer a personal approach and want their physician to be familiar with their situation, without making them feel like “one of many”. Although patients prefer repeated consultations with the same physician, they believe a switch in physicians does not negatively influence the quality of care as long as the collaboration and communication between physicians is good. In terms of communication, patients stressed the importance of physicians who truly listen to their story and make them feel at ease.

*‘It feels so good that my physician takes time for me, because my experiences with previous physicians was like ‘here are your ointments, good luck.’* – FG 2

According to patients, simple signs, including eye contact, physicians introducing themselves properly at the first consultation, and asking for patients’ preferences regarding AD care (i.e., ranging from how extensive information provision should be to the type of treatment they prefer) can facilitate in building an adequate relationship. Furthermore, patients indicated that it is very important that physicians adapt to the communicative level of the patient by, for instance, speaking in layman’s terms, taking time to explain, and actively asking if they have any questions.

### The organization of AD care

#### Need for psychosocial and medical supportive care

Patients indicated a need for increased and low-threshold psychosocial and medical supportive care. Patients understand that a physician’s time is limited and accept that this additional care is provided by other health-care workers. Patients mentioned that initial supportive care by means of extra attention and time for questions could be provided by specialized nurses.

*‘A physician has limited time during a consultation, I do understand that, but sometimes you are feeling so bad and so itchy, and everything that comes with that. It is a real problem and feels like a handicap. In situations like this, an additional appointment with a nurse who has time to listen to your story, who is in close contact with your physician, and could potentially discuss any important considerations, would be really appreciated.’* – FG2

However, the need for more profound psychosocial support provided by a psychologist or therapist varied between patients. Patients indicated that this need should be identified during their consultations and that support should be easily available when needed. Patients experienced that getting an appointment with a psychologist often takes time and effort from their side. Therefore, a direct referral to a psychologist or therapist, in particular one with experience in skin diseases, would be highly appreciated. Furthermore, patients often reported positive experiences with support from patient associations and peer contact. As such, they felt it would be useful to provide extra information about these associations in hospitals and other health-care institutions since many patients might not be aware of the existence of these groups.

#### Need for quick access to health care in AD care

As reported by patients, AD care is not always easy accessible during disease flares. Patients experienced that timing of standard consultations is not always appropriate due to the fluctuation in severity over time. As such, they expressed the need for quick access to care when their disease flares, to optimize treatment and induce remission as soon as possible. They did not report a preference for a dermatologist o,r e.g., specialized nurse in particular. However, patients mentioned several complicating factors in this process; it is difficult for them to get in touch with their physician, and the non-medical staff (e.g., administrative staff taking phone calls) is not able to understand or adequately assess the severity of their flares.

*‘My doctor tells me ‘Do call us if it gets worse’. And when I do, the assistant tells me my doctor is not available, but that I can get consultation by phone. Well, that easily takes three days and by the time I can speak to my doctor my symptoms have reduced.’* – FG 1

According to patients, clear instructions at the initial consultation on what to do in case of exacerbations, and an appropriate and personal doctor–patient relationship could facilitate quick access to care.

### The therapeutic decision-making process

#### Need for adequate, understandable, and tailored information

Patients indicated that being provided with adequate and understandable information is crucial in AD care. This information should be patient tailored since some prefer comprehensive information, while others prefer a more concise overview.

Patients highlighted several aspects in the content of this information that were crucial to them. First, patients mentioned that they are more motivated to adhere to therapy when they are aware of the underlying principles. Therefore, patients require improved disease-related information. Second, patients would like to be informed about different treatment options, their mechanism of action, and possible side effects. This should preferably be tailored to the individual patient’s situation and preferences. Additionally, patients preferred to be informed about potential next steps in the treatment process. They stated that knowing that there are other options available when necessary will put them at ease. Third, information about possible external influences on their disease such as the influence of nutrition, climate, and allergies as well as practical recommendations (e.g., daily care skin products) would be highly appreciated. On the other hand, patients indicated that repeating basic advices (e.g., advice for limited water exposure) is very bothersome, since they feel that they already “passed that stage”, which emphasizes the need for continuous patient-tailored information.

*‘I hear the same advice all the time: “you should not shower too often, not too warm and not too long; don’t use soap”. It is so annoying to hear this for the eighth time…’*—FG 2

Patients believe that underexposure of specific topics (e.g., nutritional influences) in evidence-based guidelines used by physicians might be related to a lack of evidence. Even though patients understand that there might be a lack of scientific evidence in information about these topics on the Internet, patients would appreciate physicians to understand their search for additional information. In this context, an online forum monitored by physicians would be of added value. Meetings organized by patient associations were also often considered very informative. In addition, patients considered having the possibility to access their digital medical file a positive development.

#### Varying views on the preferred role of patient and physician

Patients vary in their views regarding the preferred role of the physician and patient in the decision-making process. Some patients preferred to have a leading role and want to be informed about different treatment options by their physician, resulting in an interactive decision-making process. According to these patients, getting enough time to read and consider the provided information is essential. This autonomy would enhance shared decision-making and reduce the “trial and error” feeling experienced by some patients.

*‘I prefer it to be an interaction between me and my physician, in which I also have a say. I know myself best and I read up on specific therapies.’*—FG1

Other patients prefer their physicians to have the leading role since these patients feel that their physicians have a better overview of their situation and treatment options.

*‘I often feel like ‘Oh well, you are the professional, so tell me what would be best.’*—FG2

Generally, patients stated that physicians should get insight into each individual patient’s preference regarding their preferred role, to make appropriate decisions for each patient.

#### Decisive factors for choices within the decision-making process

Patients mentioned that several factors play an important role in their therapeutic choices. Most patients indicated that they are willing to accept side effects as long as they are reversible and if the therapy shows sufficient effectiveness on their skin disease and quality of life. However, other patients feel resistance to ‘create new problems, by treating the initial problem’.

*‘I always weigh the options: it is either one thing or the other. If I want to get rid of my eczema, I need to accept certain side effects.’***—**FG1

Patients reported that the method and frequency of administration are important in terms of feasibility, and that this should be considered by physicians as well. Additionally, patients indicated that treatment choices are also dependent on previous therapeutic experiences. They considered the physician’s recommendations and expectations regarding specific treatment options important factors as well.

Lastly, patients indicated that practical aspects including travel time and travel costs do not determine their therapeutic choices, as long as they receive high-quality care.

#### Indication for next steps within AD treatment should be patient dependent

Patients stated that a next step in their treatment is indicated when their current therapy is insufficiently effective (i.e., the disease negatively interferes with daily life) or inappropriate (e.g., due to side effects). Determining whether a certain new treatment is indicated should be patient dependent, as the impact of the disease in daily life and contributing factors differ between patients.

Most patients reported to know that therapeutic history is an important factor in their indication for new therapies (e.g., patients must have used at least 1 systemic immunosuppressant for 4 months to be eligible for dupilumab treatment [21]). Although patients have high expectations of novel therapies, they generally agree with the recommended stepwise approach when taking into account the high costs. However, they feel that physicians should be able to make some exceptions for very severe cases with a high impact on quality of life, although patients agreed that making these distinctions can be difficult.

*‘If someone is really suffering and they cannot even leave their bed and are not able to participate in normal life due to their eczema, then the physician should be able to skip a few steps in the treatment process.’*—FG 2

Additionally, patients voiced that when a patient has been treated with a systemic immunosuppressant without sufficient effectiveness, they should be able to step up more quickly the novel therapies. They stressed this could prevent a high burden for these patients.

#### Varying preferences on setting treatment goals

Most patients reported to have abstract treatment goals (e.g., increased quality of life), which are generally not discussed with their physician. Patients often feel uncertain about the effectiveness of the initiated therapy based on negative experiences with previous therapies. Consequently, they protect themselves from disappointment by not setting and discussing individual treatment goals.

*‘The physician can also not guarantee that my eczema will disappear completely. So setting big goals is an illusion for me because I find it difficult to know whether I am able to achieve that goal.’*—FG2

Other patients mentioned that they experienced more guidance and motivation when setting and evaluating individual goals. Patients agreed that when realistic goals are discussed, subsequent evaluation of these goals during consultations is essential.

## Discussion

This qualitative in-depth focus group study revealed the needs and preferences of patients regarding AD care in the era of new therapeutic options. A variety of needs and preferences were identified concerning consultations with physicians, AD care organization, and the therapeutic decision-making process. The need for a more patient-tailored approach was an overarching theme in all aspects of AD care.

With regard to consultations, patients stressed the need for autonomy in determining the impact of AD in daily life and increased recognition of the burden of disease by physicians [5]. To address these needs, supportive tools for patients to indicate the impact and disease control over a longer time period such as the Atopic Dermatitis Control Tool, validated health-related quality of life measures, or the use of personal health records (PHR) could be of added value [21–24]. Patients also indicated a need for psychosocial and medical supportive care. Studies assessing the implementation of supportive care for children with AD and their families have revealed positive results. Although applied interventions require further development, this could guide a more general implementation of supportive care [25–28].

Consistent with previous research [29, 30], our study showed that a trustful relationship with a personal approach, a physician who is truly listening to you, and the feeling of being taken seriously are essential for patients in AD care. Ha et al. stated that many doctors overestimate their ability in communication, and therefore implementation of improved formal training in communication skills in the medical curriculum could be useful [29, 31]. A well-established doctor–patient relationship might also contribute to an increased accessibility in AD care during disease flares as physicians can often intuitively determine the urgency by knowing the patient personally. However, patients reported a struggle in contacting health-care providers when needed. Several solutions to enable quick access to health care have been described, including virtual care which has rapidly been adopted because of the COVID-19 pandemic [32]. Quick accessible care showed positive outcomes and optimized resource utilization in other chronic inflammatory conditions [33, 34]. Additionally, tools for self-management including PHR [24], flare self-assessment [35] and written eczema action plans with individualized guidance on, e.g., treatment use have been shown to be helpful in AD and other allergic diseases [36–40].

To create a therapeutic ‘patient journey’ that suits the patient best, the decision-making process should be patient tailored and optimized through, e.g., consideration of the patient’s personal situation regarding eligibility and feasibility of therapies, and the patient’s right to self-determination. Patients emphasized the importance of physicians being able to make patient-dependent exceptions on applied guidelines, which are based on relatively homogeneous populations. These exemptions are particularly needed when the disease highly interferes with patients’ daily life. Previous studies have shown that therapeutic decisions should indeed be patient centered, fair, and cost-effective in the ideal situation [41]. However, this remains a practical challenge, partly because of the lack of insight into cost-effectiveness of the recently introduced therapies in daily practice [42]. A shift toward more patient-centered indication criteria incorporating the disease impact in daily life in addition to current criteria concerning therapeutic history would be highly appreciated by patients. This would facilitate in making “fair” choices, in particular for expensive therapies [8, 41]. Agencies such as the FDA and the NHS also promote patient-centered care, with improved patient access to more affordable drugs [43–45]. In addition to patient-centered care (i.e., care that is respectful of, and responsive to, individual patient’s preferences, needs, and values) [46], personalized medicine (i.e., tailoring care based on patients’ genetic information or other biomarkers) [47] is also considered important to achieve individualized care [48]. Although their sources are different and it remains unclear whether and how these two could be combined on a practical level, personalized medicine might as well contribute to individual flexibility and variability in treatment decisions, and moving away from one-size-fits-all solutions [47].

In this study we investigated a contemporary topic using data obtained from a variable sample of patients, originating from a general and an academic hospital. Our qualitative data were analyzed using multiple phases of coding alternating with consensus discussions within our multidisciplinary team, enhancing the robustness of our results [20, 49]. However, the design of this study does not allow us to draw conclusions on potential differences between patients from different medical settings. In addition to this study focusing on patients’ views, future studies investigating views of healthcare providers on different aspects of AD care might be of added value in order to further optimize AD care.

This study demonstrated that AD patients have a variety of needs and preferences regarding care. AD care in general should be patient tailored, with increased attention for the psychosocial burden, as well as better access to healthcare during disease flares. To provide patient-tailored care, therapeutic decisions should be patient dependent and the interference of the disease in daily life should be incorporated when considering indication for novel therapies. Additional needs and preferences of patients concerning, for instance, the provided information or feasibility of therapies should be taken into account in the therapeutic decision-making process, with respect for the patient’s autonomy.

## Supplementary Information

Below is the link to the electronic supplementary material.

Supplementary file1 (DOCX 24 KB)

## Data Availability

All data and materials as well as software application or custom code support their published claims and comply with field standards. The data that support the findings of this study are available from the corresponding author upon reasonable request.
