# Supplementary material for: Needs and preferences of patients regarding atopic dermatitis care in the era of new therapeutic options: a qualitative study
Source: Arch Dermatol Res. 2022 Feb 2;315(1):75–83. doi: 10.1007/s00403-021-02321-z (PMC8809237; doi:10.1007/s00403-021-02321-z)
Supplement: Supplementary file 1 — Supplementary file1 (DOCX 24 KB) [file 403_2021_2321_MOESM1_ESM.docx]

# **Supplementary Information (SI)**

**Title:** Needs and preferences of patients regarding atopic dermatitis care in the era of new therapeutic options: a qualitative study

**Journal**: Archives of Dermatological Research

**Authors**: Linde E.M. de Wijs, MD^1^, Sven van Egmond, MD, PhD^1^, Arjan C.A. Devillers, MD, PhD^2^, Tamar Nijsten, MD, PhD^1^, DirkJan Hijnen, MD, PhD^1^, Marjolein Lugtenberg PhD^1^

**Affiliations**:

^1^Department of Dermatology, Erasmus MC University Medical Center, Rotterdam, the Netherlands

^2^Department of Dermatology, Maasstad Hospital, Rotterdam, the Netherlands

**Corresponding author:** D.J. (DirkJan) Hijnen (T: 0031 10 704 0110, F: 0031107033822, E: [d.hijnen@erasmusmc.nl](mailto:d.hijnen@erasmusmc.nl))

**Online Resourse 1. Topic guide**

| **Introduction**   - Welcome - Background and aim of our study - Information on anonymity and independency - Informed consent - Questions |
| --- |
| **General experiences in AD care and impact in daily life** |
| **AD treatment**   - Experiences with healthcare providers   *Dermatologist, therapist, psychologist, nurses*   - Therapy   - Motives for commencing therapy   *Main symptoms, limitations in daily life, tipping point untenable vs tenable*   - - The decision-making process   *Information provision, role of patients*   - - Step-wise approach   *Experiences and opinions*   - - Experiences with therapy   *Information provision, pros and cons*   - Treatment goals and satisfaction |

**Online Resource 2. Characteristics of individual participants**

| **Patient** | **Focus group** | **Sex** | **Age** | **Age onset AD** | **Applied treatment** | **Disease impact in daily life*** | **Overall perceived health**** |
| --- | --- | --- | --- | --- | --- | --- | --- |
| 1 | EMC(1) | F | 27 | 0 | syst. immunosuppr. | very high | intermediate |
| 2 | EMC(1) | M | 63 | 58 | syst. immunosuppr. | low | good |
| 3 | EMC(1) | M | 41 | 0 | biologic | low | good |
| 4 | EMC(1) | F | 19 | 0 | topical therapy | high | good |
| 5 | EMC(1) | F | 47 | 0 | biologic | very low | perfect |
| 6 | EMC(1) | X | 19 | 0 | syst. immunosuppr. | very high | good |
| 7 | EMC(1) | F | 49 | 2 | syst. immunosuppr. | intermediate | good |
| 8 | EMC(1) | F | 21 | 0 | syst. immunosuppr. | high | good |
| 9 | MSH(2) | F | 62 | 1 | syst. immunosuppr. | very high | good |
| 10 | MSH(2) | F | 27 | 0 | syst. immunosuppr. | intermediate | good |
| 11 | MSH(2) | F | 28 | 0 | topical therapy | high | good |
| 12 | MSH(2) | M | 28 | 16 | syst. immunosuppr. | very high | intermediate |
| 13 | MSH(2) | F | 21 | 0 | topical therapy | very high | intermediate |
| 14 | MSH(2) | M | 57 | 1 | syst. immunosuppr. | intermediate | good |
| 15 | EMC(3) | M | 31 | 0 | trial systemic ther. | very high | bad |
| 16 | EMC(3) | M | 35 | 2 | biologic | very high | good |
| 17 | EMC(3) | F | 20 | 0 | biologic | very low | intermediate |
| 18 | EMC(3) | F | 63 | 6 | biologic | very low | good |
| 19 | EMC(3) | M | 40 | 0 | trial systemic ther. | high | intermediate |
| 20 | EMC(3) | M | 31 | 3 | topical therapy | very high | good |

* Options: 5-point Likert scale: very low, low, intermediate, high, very high

** Options: 5-point Likert scale: perfect, good, intermediate, bad, very bad

EMC: Erasmus MC Medical Center; MSH: Maasstad Hospital ; syst. immunosuppr: systemic immunosuppressants; trial systemic ther.: trial jak inhibitor; F: female; M: male, X:indeterminate

**Online Resource 3. Quotes**

| **Subthemes** | **Quote** |
| --- | --- |
| **Consultations with physicians** | |
| **Need for recognition of the physical and emotional impact of AD** | *‘My dermatologist always asks how I am instead of how my disease is, I really like that.’*  *‘I can have good conversations with my current dermatologist. It helps me to find my way through my daily struggles. Although I do not really expect this from my dermatologist, I do appreciate it.’*  *‘I have really good experiences with my dermatologist, because he was showing empathy.’*  *‘The most important part of AD care is that you feel taken seriously’*  *‘Because you know that physicians have busy schedules, you communicate briefly and professionally, and don’t feel comfortable talking about things like emotions and feelings.’* |
| **Need for increased role of patients in determining disease impact** | *‘Well, for instance, if I argued that my eczema looked much worse the day before, and I even brought a photograph with me, they might just ignore that and only look at the current state which looks much better compared to the days before. This makes you feel like you're not being taken seriously.’*  *‘You just should have an appointment at the right time, when having the worst itch and most severe pain. They are only able to see how severe and invalidating it actually is when they see you at that specific moment..’* |
| **A personal approach and adequate communication are essential** | *‘What really affected me, what I will never forget, they didn’t even look at me while speaking to me. ‘*  *‘In communication there should be understanding for the patient’s situations, things should be explained in simple language.’*  *‘Sometimes I leave the room and think: ‘I should have said this or that’. You just feel overruled by physician because of the next patient in line.’* |

| **The organization of atopic dermatitis care** | |
| --- | --- |
| **Need for psychosocial and medical supportive care** | *‘Especially patients who are alone might have the need to share their thoughts with a supportive healthcare workers, yes, I guess so..’*  *‘A nurse has knowledge on the medical part of my skin disease in particular. A psychologist has knowledge on my psychological functioning, but might not have all the insight in to my skin disease.’*  *‘In times of stress or in times of performance anxiety, my eczema gets worse, so I think that psychosocial counseling might work preventive as well.’*  *‘Maybe a nurse, who had more time than a dermatologist, could provide “aftercare” or just listen to your story.. He or she might have good ideas, like ‘try this or that’.’*  *‘It would be so nice to consult a specialized nurse who has appropriate knowledge on your disease, time for listening to your story and who is in close contact with your dermatologist.’*  *‘The added value of a patient association is that you can share your experiences, you feel understood. It also feels very trustfull because it is a private community.’*  *Pt x: ‘I have never heard about these patient associations..’*  *Pt y: ‘Well I learned about it after I found a leaflet in the waiting room at my dermatologist.’* |
| **Need for quick access to healthcare in AD care** | *‘I often feel a threshold to contact the hospital in times of a flare-up. I feel that a physician doesn’t have the time to truly listen to my story at these times..’*  *‘Back in the day, I usually had an appointment in one day after calling because of a severe flare-up. That was so nice, he knew me well and so he knew that if I asked for help, it was really serious.’* |
| **The therapeutic decision-making process** | |
| **Need for adequate and understandable information provision** | *‘When I receive information on possible treatment options, it’s mainly very extensive, at least too extensive..’*  *‘I really like that I can re-read details discussed during consultations in my personal digital patient file.’*  *‘That you gather with professionals and peers, and that things including nutrition, like “what does have an influence on your eczema and what doesn’t”, will be discussed. I would really like such meetings in which multiple aspects that could be of relevance for eczema would be discussed.’*  *‘I regret that I never heard about these relatively new drugs, that is where my struggle starts, I would rather know my future treatment options.’*  *‘Just knowing where you are standing, knowing what to expect, that is what I really appreciate.’* |
| **Varying views on the preferred role of patient and physician** | *‘I think it is an interactive process between patient and physician. I know myself, I read a lot about my medication, but I am not the one claiming to have all the knowledge, but at least I have a reliable overview on it. I prefer to discuss my considerations and questions with my physician to ultimately make a good and thoughtful choice.’*  *‘I was happy my physician made the choice, I have no idea after having tried so many ointments and therapies.’*  *‘After being informed about the options, I always ask time to consider all options and think about it.’*  *‘I really want to have input in the treatment selection process. I read a lot and I know my situation best.’*  *‘I really do not like to be in the lead in this process..’* |
| **Decisive factors for choices within the decision-making process** | *‘My decision for the treatment was mainly based on the physician’s expectation regarding the effect of the drug on my disease.’*  *‘I always think: it is either A or B. If I want to treat my eczema properly, I need to accept certain side effects.’*  *‘It also depends on your lifestyle. Every patient is different, let’s assume that you have to take pills five times a day and others once weekly, while one patient can be compliant in all situations, others can’t, for instance because of their work.’*  *‘As long as it works, additional costs don’t bother me.’*  *‘I have been driving for 2 hours per consultation in that specific hospital during the past 4 years. My eczema was so worse, I didn’t care about the long ride or costs, as long as the quality of care was that high. The impact is just too high.’* |
| **Indication for next steps within AD treatment should be patient-dependent** | *‘Sometimes you have to switch therapies because you are allowed to use some drugs only for a maximum duration.’*  *‘If someone is really suffering and not able to live a normal life, if someone is not even able to participate in daily life, I think the physician should speed up and skip steps in the treatment process.’* |
| **Varying preferences on setting treatment goals** | *‘I never know if a therapy will be effective, therefore I don’t prefer to set hopeful goals without knowing if I will be able to achieve them’*  *‘My physician and I made specific treatment goals, small steps. This was well-discussed and clear. I liked that.’*  *‘If goals are set and discussed, they should at least be evaluated later on during future consultations.’*  *‘The absence of side effects is important for being satisfied with your treatment.’* |
